# Individual variability in hydrogen-producing microbes influences the response to hydrogen supplementation on sleep quality: a randomized, double-blind, placebo-controlled, parallel study

**DOI:** 10.1038/s41598-026-52342-9

**Published:** 2026-07-27

**Authors:** Fumiko Higashikawa, Keishi Kanno

**Affiliations:** 1 Medical Center for Translational and Clinical Research, Hiroshima University Hospital, 1-2-3 Kasumi, Minami-Ku, Hiroshima, 734-8551 Japan; 2 Department of General Internal Medicine, Hiroshima University Hospital, 1-2-3 Kasumi, Minami-Ku, Hiroshima, 734-8551 Japan

**Keywords:** Hydrogen; hydrogen-producing bacteria, Sleep quality, Gut microbiome, Diseases, Health care, Medical research, Microbiology

## Abstract

Hydrogen has been reported to exert antioxidant and anti-inflammatory effects, and its potential health benefits have been investigated. However, to our knowledge, evidence regarding its impact on sleep quality in healthy individuals remains extremely limited. In addition, the influence of inter-individual variability in the gut microbiota on hydrogen efficacy has seldom been studied. In this study, we aimed to assess the impact of hydrogen-rich jelly on sleep quality and examine the influence of gut microbiota on this effect. A total of 44 healthy adults with poor sleep quality were randomized to receive either hydrogen-rich jelly or placebo jelly. No significant differences were observed between the intervention groups in the changes in sleep-related outcomes, including OSA-MA, VAS, PSQI, and STAI scores, in the overall analysis. Notably, when participants were stratified by the mean change in the VAS score for sleep quality, gut microbiota β-diversity showed apparent clustering in the hydrogen group but not in the placebo group. This cluster was explained by the differences in the relative abundance of hydrogen-producing bacteria, such as *Bacteroides*. Linear mixed-effects model analyses revealed significant interaction effects in the group × H_2_-producers in the VAS for sleep quality and mental stress, with greater improvement in participants with a lower abundance of hydrogen-producing bacteria. In conclusion, these exploratory findings raise the hypothesis that baseline gut microbiota composition, particularly microbial hydrogen-producing capacity, may modify individual responses to hydrogen supplementation. This hypothesis warrants confirmation in larger studies to explore its implications for microbiome-informed stratification in future hydrogen intervention research.

*Clinical trial registration*: This clinical trial was registered with the University Hospital Medical Information Network Clinical Trial Registry on 27/12/2023 (UMIN-CTR, UMIN000053237).

**Supplementary Information:**

The online version contains supplementary material available at 10.1038/s41598-026-52342-9.

## Introduction

In recent years, it has become increasingly clear that molecular hydrogen has antioxidant, anti-inflammatory, and anti-apoptotic properties. Notably, molecular hydrogen is extremely small and can penetrate cellular membranes to reach mitochondria. Hydrogen reduces oxidative stress by scavenging hydroxyl radicals and modulates inflammation through pathways such as NF-κB inhibition and Nrf2 activation. Ohsawa et al. have demonstrated that hydrogen can selectively eliminate hydroxyl radicals, considered the most harmful among reactive oxygen species (ROS), and suppress brain injury induced by focal ischemia–reperfusion^1^. Hydrogen-rich water increased superoxide dismutase (SOD) activity and significantly decreased malondialdehyde content in the ischemia–reperfusion phase by activating the Nrf2/ARE signaling pathway in rats^2^. In addition, molecular hydrogen inhibits the phosphorylation of NF-κB and significantly reduces inflammation after stroke in diabetic rats^3^. To date, many studies have reported in animal models and clinical studies that the administration of molecular hydrogen by inhalation or as hydrogen-rich water may have beneficial effects on central nervous system diseases, cardiovascular diseases, cancer, obesity and metabolic diseases, infections, respiratory diseases, gastrointestinal diseases, inflammatory diseases, and/or healthful longevity^4–7^. Accumulating evidence suggests that excessive oxidative stress is closely associated with sleep disturbances, and reducing oxidative stress may improve sleep quality^8^. Indeed, recent studies have reported that exogenous hydrogen administration enhances sleep consolidation in mice^9^ and improves subjective sleep quality in patients with long-COVID or obesity^10,11^. However, little is known about the effect of hydrogen on sleep quality in healthy individuals, and whether its antioxidant properties translate into sleep benefits for this population remains to be elucidated.

Because hydrogen is highly volatile and prone to rapid dissipation, special attention must be paid to its storage, packaging, and delivery methods. To date, there is no widely established or standardized approach for administering hydrogen in clinical or practical settings. Although hydrogen-rich water is commercially available, there are currently no clear regulatory standards for residual hydrogen concentration, making its consistency and efficacy difficult to assess. Moreover, the water solubility of hydrogen is low (1.57 mg/L), which limits its dosage^5^. Another option for administering hydrogen is either inhalation of hydrogen gas or oral intake of hydrogen-rich jelly, as used in this study. Hydrogen-rich jelly is a new delivery method that can hold a higher concentration of hydrogen than hydrogen-rich water. However, in fact, adequate dosages for efficacy have not been fully clarified, although safety issues may not be a concern^5,12^.

It is also known that gut microbes generate hydrogen during their metabolic activities, such as carbohydrate fermentation, and may subsequently be utilized by cross-feeding microbes^13,14^. Bacteria-derived hydrogen passes through the intestinal mucosa and circulates throughout the body via the bloodstream^15^. However, there is considerable inter-individual variability in gut microbiota composition, implying that hydrogen production varies widely from person to person. Although the optimal amount of hydrogen production or the ideal balance between hydrogen production and consumption by hydrogenotrophic microbes has not yet been elucidated, individual variability in hydrogen production may be associated with an increased risk of human disease. Parkinson’s disease^16,17^ and rheumatoid arthritis^18^ have been implicated in the reduction of gut bacteria-derived hydrogen^15^. However, it is difficult to find studies that consider the inter-individual variability of hydrogen-producing bacteria in the gut to assess the efficacy of exogenous hydrogen on various health-related conditions. We hypothesized that hydrogen supplementation would reduce systemic oxidative stress, thereby improving sleep quality, and that the abundance of hydrogen-producing bacteria affects the effectiveness of hydrogen supplementation. Hence, we conducted a randomized clinical trial to assess the effects of hydrogen-rich jelly on sleep quality and oxidative stress in healthy participants with insufficient sleep quality. The primary outcome was defined as the change in subjective sleep quality assessed by the Oguri–Shirakawa–Azumi Sleep Inventory for Middle-Aged and Aged (OSA-MA). Secondary outcomes included changes in other sleep and stress questionnaires (VAS, PSQI, and STAI), oxidative stress markers, and gut microbiota composition, allowing us to examine whether inter-individual variability in gut microbiota modifies the efficacy of hydrogen.

## Materials and methods

### Materials

Hydrogen-rich jelly included 0.3 mg hydrogen, 9.81 g water, 0.17 g gelling agent, Clearagar (MN#150, AOBA KASEI CO., LTD., Sendai, Miyagi, Japan), 0.01 g sucrose esters, and 0.01 g acesulfame potassium in an aluminum pouch stick package. As this is a commercially available product, the hydrogen concentration and its stability within the aluminum pouch over its shelf-life (24 months) under standard storage conditions (at room temperature in a cool, dry place away from direct sunlight) are standardized and guaranteed by the manufacturer’s strict quality control. The placebo jelly contained air instead of hydrogen. To ensure blinding, the placebo jelly was perfectly matched to the hydrogen jelly, being completely identical in packaging appearance, texture, taste, and smell. The participants consumed three packages daily, resulting in a total daily intake of 0.9 mg of hydrogen or air. The dosage (0.3 mg per administration) is comparable to that used in previous clinical studies demonstrating that a single oral administration of hydrogen-rich water increases breath hydrogen concentration^19,20^. Hydrogen and placebo jellies were provided by Shinryo Corporation (Fukuoka, Japan).

### Participants

Participants were recruited through advertisements in Hiroshima. Healthy adults with continuous dissatisfaction with sleep quality aged between 20 and 75, and the average standardized score of five factors of OSA-MA (Oguri–Shirakawa–Azumi Sleep Inventory for Middle-Aged and Aged)^21^ was below 45, were the inclusion criteria. The exclusion criteria were as follows: those under treatment for chronic disease; those who were taking medication or dietary supplements that may impact the study results, such as antioxidant reagents; those who had participated in another clinical trial within the past three months; those who were pregnant or breastfeeding; and those who were judged as ineligible by investigators. The borderline score of 45 for the OSA-MA score was not disclosed to the participants in advance to prevent them from intentionally changing their answers.

### Study design

This randomized, double-blind, placebo-controlled, parallel-group clinical trial was conducted at Hiroshima University Hospital between January and May 2024. A total of 44 participants who met the inclusion and exclusion criteria, as determined by the principal investigator, were randomly assigned in a 1:1 ratio to either the hydrogen or placebo group. Randomization was performed using a computer-generated list, with stratification by sex and block randomization (block size: 4). The investigator responsible for generation of the random allocation sequence and group assignment played no role in the evaluation of outcomes. The principal investigator, treating physicians, and clinical personnel remained blinded to the participant allocation throughout the study.

The primary outcome was the change in the OSA-MA score over an 8-week period. Secondary outcomes included changes in the visual analog scale (VAS) scores for sleep quality, mental stress, and physical stress; PSQI score; State-Trait Anxiety Inventory (STAI); frequency of bowel movement; oxidative stress and antioxidant markers; and 16S rRNA amplicon sequencing of the gut microbiome. The safety of the hydrogen-rich jelly was assessed throughout the study period.

The participants received three packages of either hydrogen or placebo jelly daily, according to their assigned treatment. They were instructed to consume the jelly directly from the package and swallow it quickly after opening. In addition, they had to take jelly three times a day at intervals longer than 2 h.

The participants were instructed as follows: (1) avoid overeating and maintain their usual lifestyle, including exercise; (2) record daily entries in their study diary, including whether they consumed the placebo/hydrogen-rich jelly, their health conditions, any medications or dietary supplements taken, and the number of spontaneous bowel movements; (3) keep a detailed record of all foods and beverages consumed, including snacks and alcohol, for three days before each clinical visit; (4) do not start taking any new dietary supplements after the study has begun; and (5) do not donate blood during the study period.

The participants’ compliance rates were evaluated based on their daily records. Compliance was calculated as the percentage of consumed jelly packages out of the total amount (168 packages over 8 weeks). To avoid overestimation, any missing daily diary entries were strictly treated as “not consumed”. Any adverse effects that occurred during the study were recorded in a questionnaire at the clinical visit and in the participants’ diaries, and assessed using the Common Terminology Criteria for Adverse Events (CTCAE) version 5.0.

At the end of the 8-week intervention period, blinding success was assessed by asking participants to guess their assigned treatment (hydrogen-rich jelly, placebo jelly, or “don’t know”).

### Questionnaires for sleep quality

The OSA-MA is a self-report questionnaire designed to assess subjective sleep quality in middle-aged and older adults^21^. It evaluates five key factors: Factor 1, Sleepiness upon awakening; Factor 2, Initiation and maintenance of sleep; Factor 3, Frequency of dreaming; Factor 4, Feeling of refreshment; and Factor 5, Sleep duration. The OSA-MA is widely used in clinical and research settings in Japan to monitor sleep patterns, screen for sleep disturbances, and evaluate the effects of interventions in aging populations. Higher OSA-MA scores indicate better sleep quality.

Visual analog scales (VAS) were used to assess the level of subjective symptoms related to sleep quality, mental stress, and physical fatigue in the participants. Participants were asked to mark their feelings on a 100 mm line, ranging from 0 to 100. The VAS indicators were as follows: for sleep quality, 0 represented “the best sleep quality imaginable” and 100 represented “the worst sleep quality imaginable”; for mental stress, 0 represented “no mental stress at all” and 100 represented “the worst mental stress imaginable”; for physical fatigue, 0 represented “no physical fatigue at all” and 100 represented “the worst physical fatigue imaginable” during the past week up to the day they completed the questionnaire.

The Pittsburgh Sleep Quality Index (PSQI) is a widely used self-report questionnaire that assesses sleep quality and disturbances over a 1-month period, with a global score range of 0–21; higher scores indicate poorer sleep quality^22^. The PSQI is commonly used in both clinical and research settings to screen for sleep problems and to evaluate treatment outcomes.

The STAI is designed to measure two types of anxiety: (1) state anxiety—how a person feels right now, in the moment, and (2) trait anxiety—a person’s general tendency to experience anxiety across situations^23^. The STAI is widely used in psychology and health research to assess anxiety levels, evaluate the impact of stress, and monitor changes due to interventions or life events. Higher scores indicate elevated anxiety levels.

Each questionnaire was recorded at weekly intervals (0-, 1-, 2-, 3-, 4-, 5-, 6-, 7-, and 8-week).

### 16S rRNA amplicon sequencing for gut microbiome

Fecal samples were collected both before and after the intervention. Participants were instructed to avoid stool sampling within 7 days of antibiotic use to minimize acute fluctuations in microbiome composition while maintaining study feasibility. No participants reported antibiotic use immediately before stool sampling. Fecal total genomic DNA was extracted using a Fecal DNA Extraction Kit (NIPPON GENE Co., Ltd., Tokyo, Japan).

Amplicon sequencing of the 16S rRNA gene (V3–V4 region) was performed using the primers 341F (5′-CCTACGGGNGGCWGCAG-3′) and 806R (5′-GACTACHVGGGTATCTAATCC-3′) with overhang adapters. Library preparation was performed using the Nextera XT Index Kit (Illumina, Inc., San Diego, CA, USA) according to the manufacturer’s instructions. Sequencing was conducted on an Illumina MiSeq platform using the MiSeq Reagent Kit v3 (2 × 300 bp paired-end).

The sequence data were processed using QIIME2 (version 2024.10)^24^. Paired-end reads were quality-filtered and denoised using the DADA2 plugin (qiime dada2 denoise-paired) with trim-left-f = 19, trim-left-r = 20, trunc-len-f = 270, trunc-len-r = 210, max-ee-f = 5.0, max-ee-r = 5.0, and n-threads = 4, generating an amplicon sequence variant (ASV) feature table and representative sequences; DADA2’s default chimera removal was applied. Denoising statistics were summarized to confirm read retention and merging performance across samples. No additional taxonomic filtering was applied to the resulting ASVs.

Taxonomic assignment was performed using a Naïve Bayes classifier trained on the SILVA 138 database (Silva 138 99% OTUs full-length sequences)^25,26^. Alpha and beta diversity metrics were calculated using QIIME2 diversity plugin. For diversity analyses, samples were rarefied to 5000 sequences per sample using qiime diversity core-metrics-phylogenetic. Beta diversity was assessed using weighted UniFrac distances, and principal coordinate analysis (PCoA) plots were generated using Emperor. Furthermore, for specific taxonomic comparisons of known hydrogen-producing bacteria, taxa with a relative abundance of < 0.005 were excluded.

### Oxidative stress and antioxidative markers

We analyzed the urinary oxidative stress markers, 8-hydroxy-2′-deoxyguanosine (8-OHdG) and isoprostanes. We also measured oxidative stress markers, antioxidant markers, pro-oxidant metals, and lipids in the serum, including lipid peroxidation (LPO), coenzyme Q10 oxidation ratio, serum total antioxidant status (STAS), water-soluble antioxidants (vitamin C, folate, vitamin B_12_, and uric acid), lipid-soluble antioxidants (tocopherol isoforms, lutein + zeaxanthin, β-cryptoxanthin, lycopene, α-carotene, β-carotene, and vitamin A), Fe, Cu, total cholesterol, and triglycerides. All markers were assessed at 0- and 8-week. The measurements were outsourced to NIKKEN SEIL Co., Ltd. (Shizuoka, Japan).

### Statistical analysis

The sample size was calculated as 21 for one group when set to the following conditions: the expected difference between groups in the 8-week change is 2.5, and the SD is 2.0 of the average of five factors of OSA-MA; 1% alpha error adjusted multiplicity by the Holm method; 90% power. We decided on a sample size of 44 for the two groups, considering the possibility of dropouts. The expected value above was determined with reference to similar studies^27^, although the criteria and intervention periods differed.

**Fig. 1:**
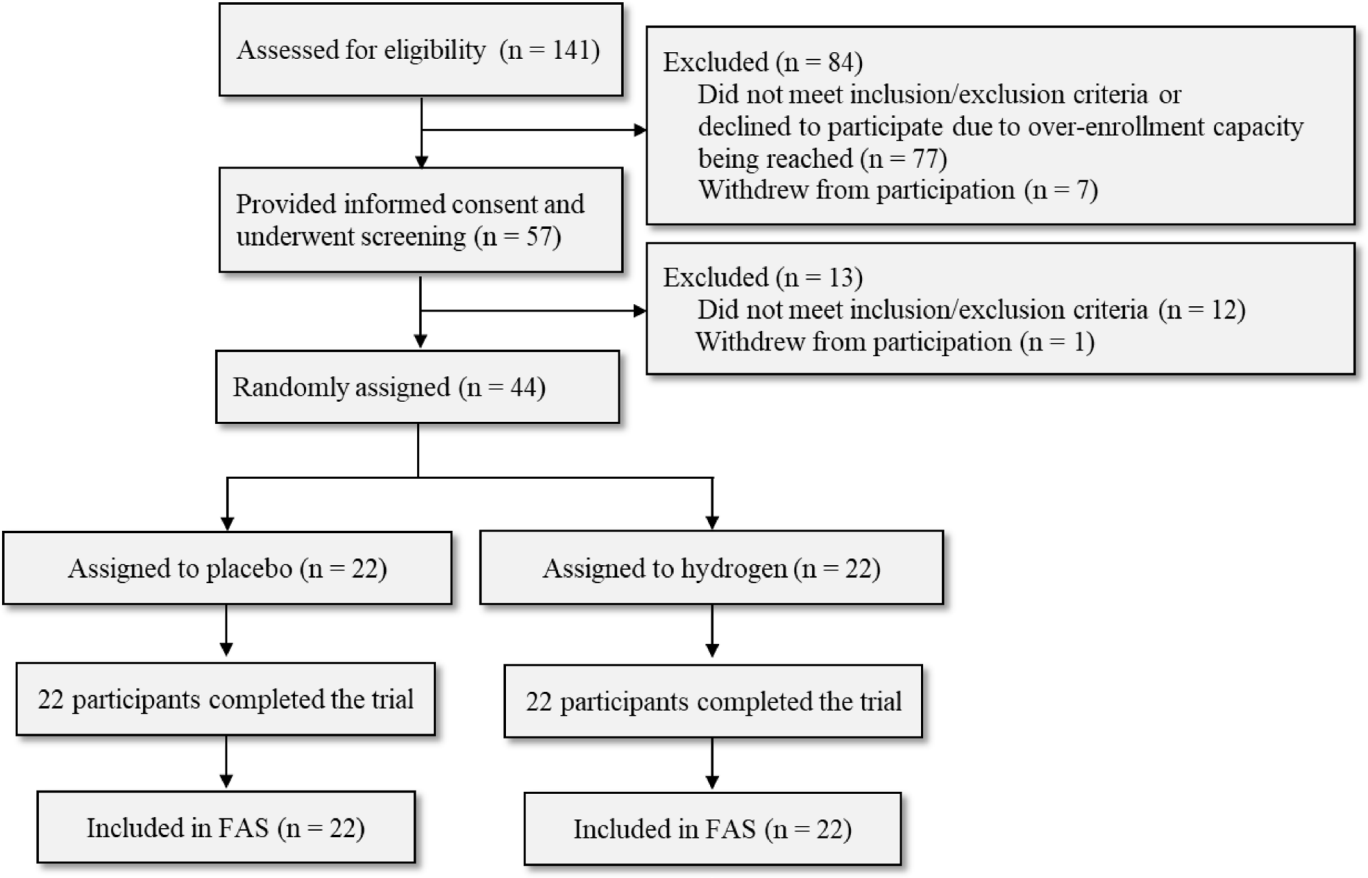
The subject flow.

As there were no dropouts from the study and no cases that violated the FAS, all randomized cases were included in the final analysis (Fig. 1). We compared the changes in each outcome during the 8-week intervention period between groups using either Student’s *t*-test (after logarithm transformation, if applicable) or the Mann–Whitney U test, depending on the data distribution. For pre- and post-intervention comparisons, either a paired t-test or a paired-sample Wilcoxon signed-rank test was used, depending on the data distribution. Group differences in bacterial relative abundances in the gut microbiota were assessed using the Kruskal–Wallis test, followed by post-hoc pairwise comparisons (between the subgroups of improvement status within each intervention group) with the Mann–Whitney U test, adjusted using the Holm correction. Group differences in β-diversity were tested by PERMANOVA with 999 permutations using weighted UniFrac distance matrices. Effect size was reported as R^2^. Homogeneity of multivariate dispersion was assessed using PERMDISP with 999 permutations. When pairwise tests were performed, *p*-values were adjusted for multiple comparisons using the Benjamini–Hochberg procedure and are reported as q-values. To determine the influence of individual variability in the gut microbiome, linear mixed-effects models (LMMs) were applied to the repeatedly measured main outcomes. The fixed effects included group, time, ‘H_2_-Producers’ (combined abundance of *Bacteroides*, *Escherichia, Ruminococcus,* and *Clostridium *sensu stricto 1) as a continuous covariate, group × time, and group × H_2_-Producers. Time was modeled as a categorical factor including all nine measurement points (baseline and weeks 1–8). The LMMs were used to evaluate overall fixed effects and interactions; therefore, additional post-hoc pairwise comparisons were not performed. We did not include time × H_2_-Producers and group × time × H_2_-Producers in the analysis to reduce model complexity and avoid overparameterization, given our sample size. This genus-level sum was used as an indirect proxy for hydrogen-producing potential and does not represent a direct measure of in vivo hydrogen production.

For categorical data analysis, Fisher’s exact test was used to compare groups for safety assessment. Data are expressed as mean ± SD in tables or mean ± SE in Supplementary Figure S1. Statistical analyses were performed using IBM SPSS Statistics Version 30.0 (IBM Corp., Armonk, NY, USA), with statistical significance set at *p* < 0.05.

## Results

### Population characteristics

Forty-four participants were enrolled and randomized, and all completed the study (Fig. 1). Table 1 summarizes the baseline characteristics of the participants. During the intervention period, there were no differences in calorie intake between the groups, as indicated by the dietary records. The intake percentages of jelly were 95.4% in the placebo group and 95.2% in the hydrogen group throughout the study period, without a statistically significant difference between the groups.

**Table 1:** Baseline characteristics of the study participants.

	Placebo (n = 22)	Hydrogen (n = 22)	*p* value
Age (y)	52.9 ± 12.5 (29–75)	60.2 ± 13.6 (22–74)	0.072
Male/Female	8/14	8/14	–
Height (cm)	160.4 ± 6.1	161.6 ± 8.5	0.62
Body weight (kg)	56.5 ± 12.4	60.7 ± 14.7	0.307
BMI (kg/m^2^)	21.8 ± 3.7	23.0 ± 4.1	0.297
Body fat percentage (%)	26.0 ± 7.2	28.0 ± 8.9	0.413
Systolic blood pressure (mmHg)	112.9 ± 16.8	119.0 ± 20.5	0.287
Diastolic blood pressure (mmHg)	69.8 ± 10.6	75.7 ± 12.2	0.092
Heart rate (beats per min)	70.0 ± 9.6	70.8 ± 7.6	0.748
White blood cell count (× 10^3^/mL)	5.29 ± 1.43	5.57 ± 1.70	0.549
Red blood cell count (× 10^6^/mL)	4.75 ± 0.45	4.71 ± 0.42	0.773
Hemoglobin (g/dL)	14.4 ± 1.1	14.5 ± 1.4	0.838
Hematocrit (%)	45.4 ± 3.7	45.9 ± 3.9	0.659
Platelet count (× 10^4^/mL)	26.0 ± 3.5	25.8 ± 6.3	0.886
AST (IU/L)	25.4 ± 7.5	23.9 ± 7.4	0.522
ALT (IU/L)	23.0 ± 13.3	24.8 ± 22.9	0.743
γ-GTP (IU/L)	26.8 ± 13.6	34.9 ± 37.2	0.346
LDH(IU/L)	195 ± 27	199 ± 27	0.62
Choline esterase(IU/L)	350 ± 117	350 ± 53	0.992
Alkaline phosphatase(IU/L)	70.6 ± 23.4	75.3 ± 29.2	0.561
Amylase (IU/L)	90.1 ± 26.9	91.8 ± 39.0	0.869
Total protein (g/dL)	7.40 ± 0.46	7.38 ± 0.41	0.891
Total bilirubin (mg/dL)	0.61 ± 0.24	0.74 ± 0.27	0.095
Albumin (g/dL)	4.46 ± 0.24	4.54 ± 0.26	0.309
Uric acid (mg/dL)	5.11 ± 1.34	4.80 ± 1.59	0.477
Blood urea nitrogen (mg/dL)	13.2 ± 3.4	13.5 ± 2.8	0.752
Creatinine (mg/dL)	0.69 ± 0.12	0.72 ± 0.16	0.465
eGFR (ml/min/1.73m2)	97.3 ± 19.9	91.5 ± 23.1	0.37
Total cholesterol (mg/dL)	229 ± 40	231 ± 32	0.842
LDL cholesterol (mg/dL)	136 ± 33	138 ± 29	0.877
HDL cholesterol (mg/dL)	70.5 ± 18.2	74.3 ± 17.3	0.48
LDL/HDL ratio	2.06 ± 0.70	1.99 ± 0.75	0.755
Triglycerides (mg/dL)	108.1 ± 96.3	95.3 ± 38.3	0.563
Fasting blood glucose (mg/dL)	98.4 ± 8.7	106.0 ± 15.9	0.053

Data are expressed as mean ± SD.

No statistically significant differences were observed between the groups.

Regarding the assessment of blinding success, 7 of the 22 participants in the hydrogen group correctly guessed their allocation (8 guessed placebo, 7 answered “don’t know”). In the placebo group, 11 of the 22 participants correctly guessed their allocation (5 guessed hydrogen, 6 answered “don’t know”). Overall, only 18 out of 44 participants (40.9%) correctly identified their assigned group, confirming that the double-blind conditions were successfully maintained throughout the study.

### Changes in the main outcomes for the overall study population

Regarding the primary outcome measure, the OSA-MA scores (e.g., Factor 2: Initiation and maintenance of sleep) significantly improved from baseline in both the placebo and hydrogen groups over the 8-week intervention period. Similarly, within-group analyses revealed that other subjective sleep measures, including the VAS scores for sleep quality, mental stress, and physical fatigue, were significantly improved in both groups. Although these within-treatment improvements tended to be more pronounced in the hydrogen group, no statistically significant differences were observed between the groups (Table 2). In the comparison between pre- and post-intervention in each group, no significant shifts were observed in the gut microbiome 16S rRNA analyses in either group.

**Table 2:** Changes in main outcomes during the intervention period in overall participants.

	Placebo (n = 22)			Hydrogen (n = 22)		
	Week 0	Week 8	Change in 8 weeks	Week 0	Week 8	Change in 8 weeks
OSA-MA factor 1 (sleepiness upon awakening)	41.4 ± 8.0	44.8 ± 6.5†	3.4 ± 5.9	40.7 ± 7.1	43.2 ± 8.7	2.5 ± 7.7
OSA-MA factor 2 (initiation and maintenance of sleep)	34.2 ± 10.6	40.7 ± 9.8†	6.4 ± 10.9	34.5 ± 6.8	40.0 ± 6.1†††	5.5 ± 7.9
OSA-MA factor 3 (frequency of dreaming)	39.2 ± 11.9	42.5 ± 11.8	3.3 ± 11.5	44.0 ± 12.0	43.9 ± 12.0	− 0.1 ± 12.5
OSA-MA factor 4 (feeling of refreshment)	40.1 ± 5.7	42.3 ± 8.3	2.2 ± 7.1	39.0 ± 6.4	41.9 ± 6.4	2.9 ± 9.4
OSA-MA factor 5 (sleep duration)	43.1 ± 11.0	46.0 ± 8.5	2.9 ± 9.7	40.8 ± 8.1	43.9 ± 8.2	3.1 ± 9.0
VAS score for sleep quality	66.9 ± 13.4	56.4 ± 20.5†	− 10.5 ± 22.0	70.8 ± 14.5	54.9 ± 22.6 †††	− 15.9 ± 18.8
VAS score for mental stress	63.7 ± 18.0	54.3 ± 23.2†	− 9.3 ± 19.5	67.5 ± 16.2	52.5 ± 25.9†††	− 15.0 ± 20.9
VAS score for physical fatigue	68.5 ± 15.1	51.4 ± 22.9†††	− 17.1 ± 20.8	68.0 ± 19.2	54.0 ± 23.8 †	− 14.0 ± 26.0
PSQI score	9.32 ± 3.06	6.86 ± 2.80†††	− 2.45 ± 2.32	8.91 ± 2.91	7.36 ± 3.17†††	− 1.55 ± 1.79
STAI state	51.8 ± 8.9	47.6 ± 9.0†	− 4.2 ± 7.2	54.8 ± 9.2	51.6 ± 10.9	− 3.1 ± 8.2
STAI trait	50.8 ± 8.9	48.8 ± 9.4	− 2.0 ± 4.6	54.7 ± 11.1	52.1 ± 10.5 †	− 2.6 ± 5.7

Data are expressed as mean ± SD.

Statistically significant differences between groups were determined using the Student’s t-test or Mann–Whitney U test, depending on the data distribution.

† and ††† indicate statistically significant differences versus the corresponding baseline, determined by paired *t*-test or paired-sample Wilcoxon signed-rank test at *p* < 0.05 and *p* < 0.005, respectively.

### Distinct β-diversity distribution based on intervention responsiveness

**Fig. 2:**
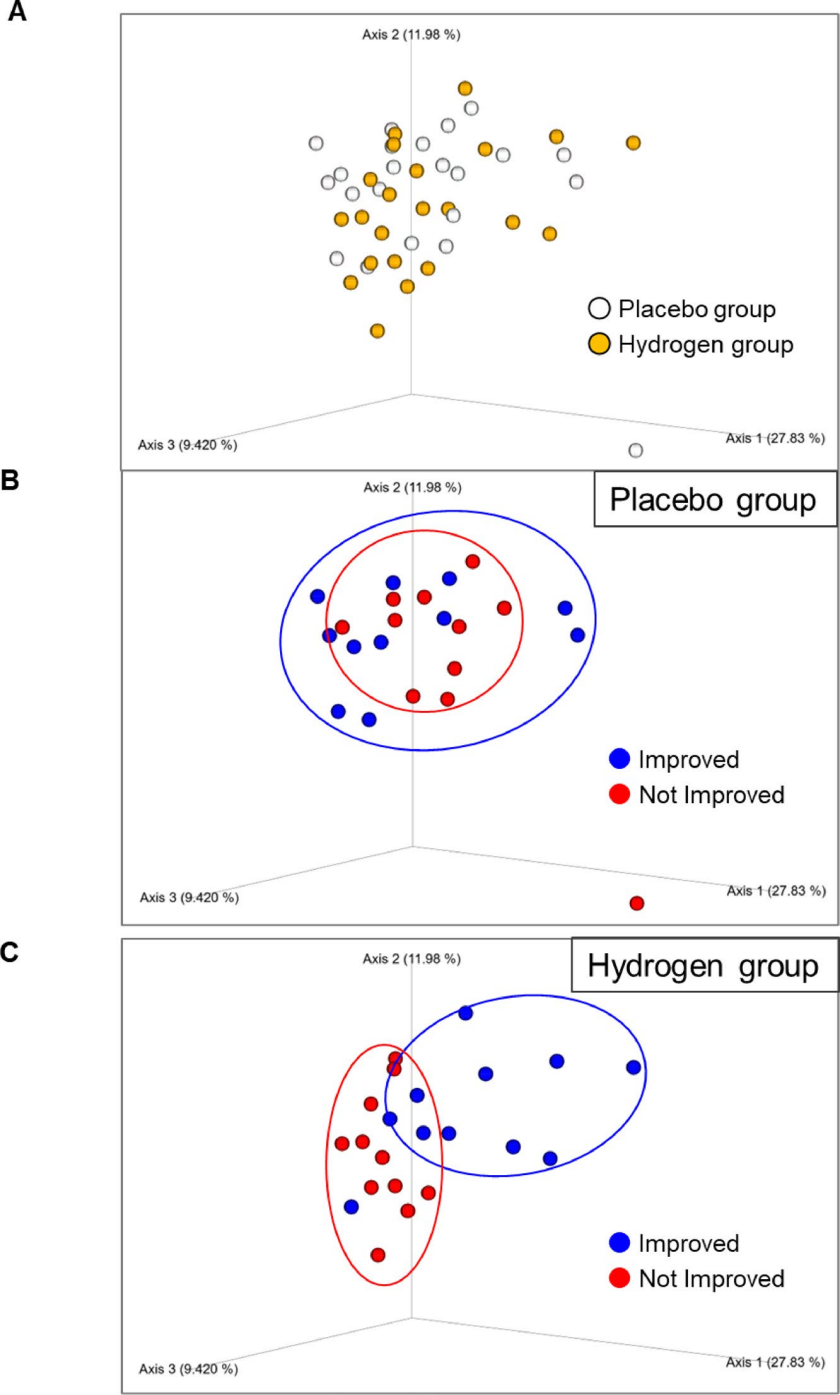
Principal coordinate analysis (PCoA) based on Weighted UniFrac distances of the gut microbiome before the intervention. (**A**) Placebo and hydrogen groups; (**B**) (**C**) placebo and hydrogen groups, respectively. ‘Improved’ indicates that the mean change in VAS for sleep was lower than the median; ‘Not improved’ indicates that the mean change was higher than the median. The median was calculated for all participants, regardless of group assignment.

Figure 2 shows the results of the Weighted UniFrac analysis, which reflects β-diversity based on both evolutionary distance and abundance of the gut microbiota at the pre-intervention (baseline) stage. The distributions of both groups were similar (Fig. 2A). As shown in Table 2, the VAS scores for the three items were reduced in both groups, and the overall median of the mean changes in the VAS score for sleep quality during the intervention period was -9.9 mm. For exploratory and visualization purposes, we divided the participants into two categories: the ‘improved’ subgroup with a mean change in VAS score for sleep quality < − 9.9 and the ‘not improved’ subgroup with a mean change in VAS score ≥ − 9.9, to determine whether any relationship exists between the change in sleep quality by hydrogen supplementation and gut microbiome characteristics. This dichotomization was used to visualize overall trends; our interpretations regarding the relationship between sleep quality improvement and gut microbiome characteristics are based on the continuous-variable LMMs and correlation analyses presented in subsequent sections. The samples were evenly distributed without category-specific clustering in the placebo group (Fig. 2B). In contrast, the distribution in the hydrogen group showed a distinct separation between ‘improved’ and ‘not improved’ (Fig. 2C), indicating differences in community composition among these four subgroups (PERMANOVA on weighted UniFrac distances, pseudo-F = 1.64, *p* = 0.033; 999 permutations, n = 44). The corresponding effect size was R^2^ = 0.109. Homogeneity of multivariate dispersion did not differ significantly among the four subgroups (PERMDISP F = 1.55, *p* = 0.214; 999 permutations). Pairwise comparisons indicated significant differences between ‘Hydrogen group -not improved’ and ‘Hydrogen group -improved’ (*p* = 0.004, *q* = 0.024), but not for the other pairs.

### Individual variability in hydrogen-producing bacteria influences the changes in sleep quality

**Fig. 3:**
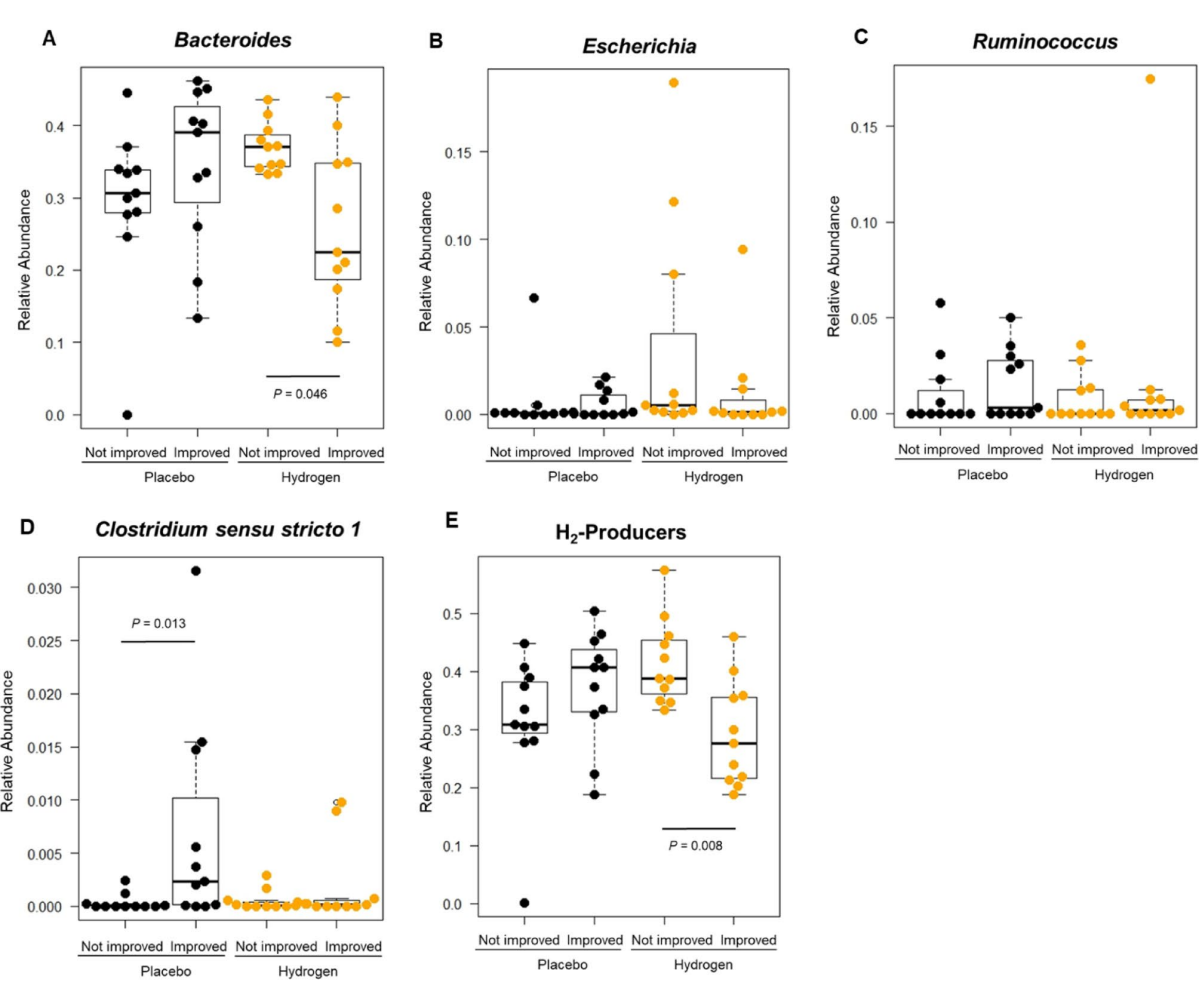
Differences between the two subgroups, ‘improved’ or ‘not improved’, in the relative abundance of hydrogen-producing bacteria at baseline. ‘Improved’ indicates that the mean change in VAS for sleep was lower than the median; ‘Not improved’ indicates that the mean change was higher than the median. The median was calculated for all participants, regardless of group assignment. The Mann–Whitney U-test was applied. (**A**) *Bacteroides*; (**B**) *Escherichia*; (**C**) *Ruminococcus*; (**D**) *Clostridium sensu strict*o *1*; (**E**) H_2_-Producers (the sum of A to D).

Next, we compared the relative abundance of gut microbes at the genus level between the ‘improved’ and ‘not improved’ subgroups in each intervention group. Figure 3 shows that major hydrogen-producing bacteria occupied a relatively higher abundance in the gut, excluding lower abundance < 0.005 from known hydrogen-producing bacteria^15,28–31^. Among them, *Bacteroides* showed a significant difference between the subgroups of improvement status in the hydrogen group (*p* = 0.046), but not in the placebo group (Fig. 3A). Moreover, the combined relative abundance of ‘H_2_-Producers’ (*Bacteroides* + *Escherichia* + *Ruminococcus* + *Clostridium *sensu stricto* 1,* used as an indirect proxy for hydrogen-producing potential) showed more obvious distinctions between the subgroups in the hydrogen group (*p* = 0.008, Fig. 3E), although *Escherichia*, *Ruminococcus,* and *Clostridium *sensu stricto* 1* did not show a significant difference (Fig. 3B, C, D). The major hydrogen-consuming bacteria, *Desulfovibrio* and *Methanobrevibacter*, were detected in only 9 and 3 participants, respectively, and had low abundance (< 0.01 and < 0.005, when detected, respectively), without differences depending on the improvement status (data not shown).

The LMM with the relative abundance of ‘H_2_-Producers’ as a covariate showed significant interaction between group and ‘H_2_-Producers’ in OSA-MA Factor 2, VAS score for sleep quality, and VAS score for mental stress, and a trend toward significance in OSA-MA Factor 4 and VAS score for physical fatigue (Table 3). Additionally, in a sensitivity analysis further adjusted for age, the group × H_2_-producers interaction for the VAS score for sleep quality remained statistically significant (*p* = 0.009).

**Table 3:** Impact of the abundance of hydrogen-producing bacteria on sleep quality improvement by hydrogen-rich jelly supplementation.

	Group	Time	H_2_-producers	Group*Time	Group*H_2_-Producers
OSA-MA factor 1 (sleepiness upon awakening)	0.519	0.117	0.598	0.614	0.360
OSA-MA factor 2 (initiation and maintenance of sleep)	0.023*	0.043*	0.785	0.176	0.012*
OSA-MA factor 3 (frequency of dreaming)	0.131	0.791	0.260	0.974	0.155
OSA-MA factor 4 (feeling of refreshment)	0.073	0.053	0.726	0.693	0.055
OSA-MA factor 5 (sleep duration)	0.898	0.462	0.001***	0.854	0.900
VAS score for sleep quality	0.013*	0.012*	0.077	0.732	0.008**
VAS score for mental stress	0.008**	0.070	0.093	0.050	0.005**
VAS score for physical fatigue	0.086	0.022*	0.198	0.628	0.079
PSQI score	0.611	0.001***	0.786	0.755	0.548
STAI state	0.801	0.096	0.555	0.558	0.911
STAI trait	0.952	0.236	0.646	0.721	0.749

Data are *p-values* from a linear mixed-effects model (LMM). The relative abundance of H_2_-Producers (the sum of the four hydrogen-producing bacterial genera shown in Fig. 3) at baseline was included as a covariate to adjust for its potential influence on the outcomes.

*, **, *** indicate statistical significance at *p* < 0.05, *p* < 0.01, *p* < 0.005, respectively.

### The relationship between the gut microbiota at baseline and sleep quality improvement

**Fig. 4:**
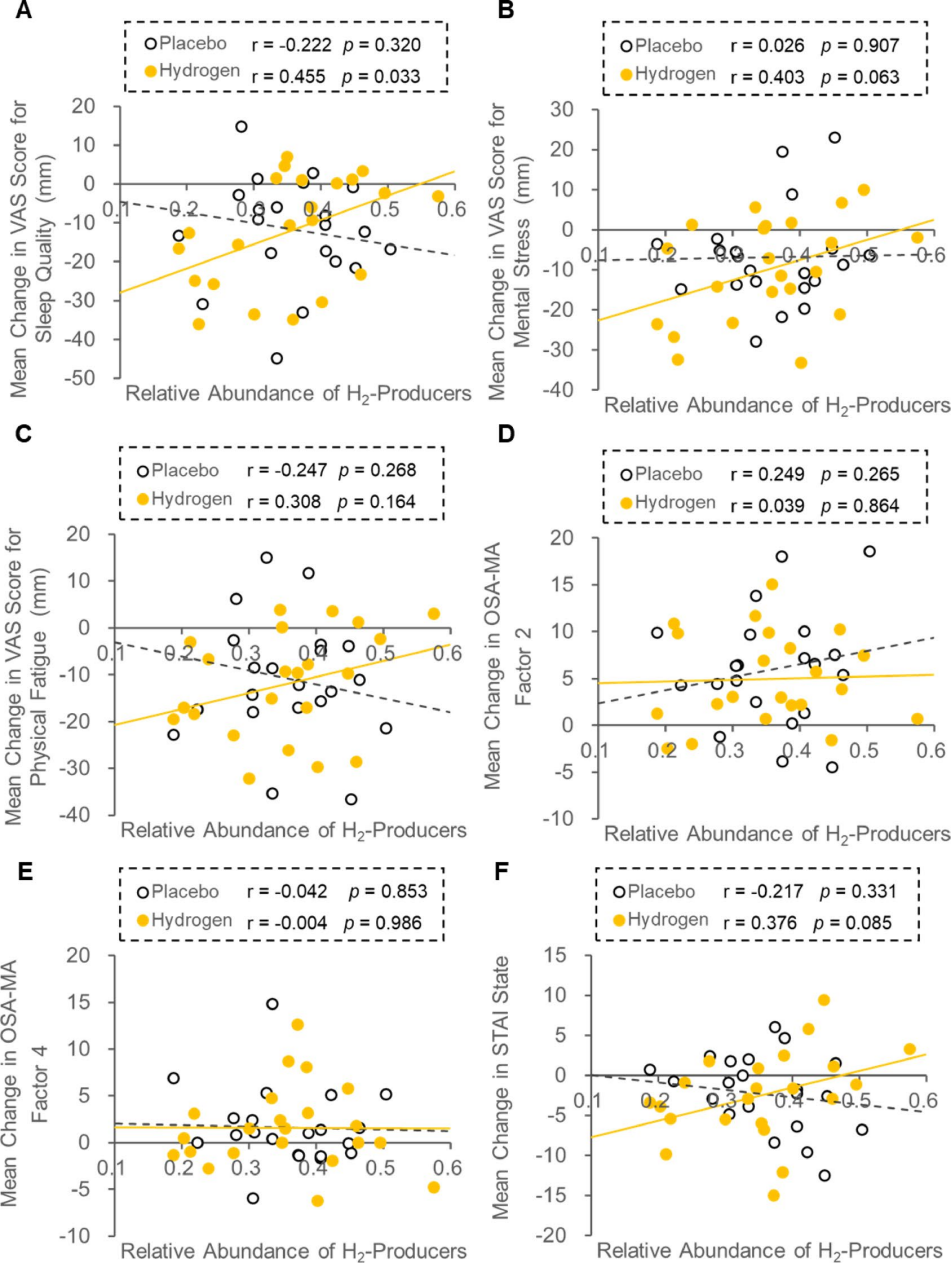
Correlation between the change in the main outcomes and the relative abundance of H_2_-Producers (the sum of the four hydrogen-producing bacterial genera shown in Fig. 3) at baseline. (**A**) VAS score for sleep quality; (**B**) VAS score for mental stress; (**C**) VAS score for physical fatigue; (**D**) OSA-MA factor 2; (**E**) OSA-MA factor 4; (**F**) STAI state. Pearson correlation coefficients (r) and corresponding *p*-values are shown in each plot.

To determine the direction of the influence on the outcome changes by hydrogen-producing bacteria, we checked the correlation between each outcome (detected significant group*H_2_-producer interaction or its tendency) and the relative abundance of ‘H_2_-Producers’ in the placebo and hydrogen groups separately (Fig. 4). Pearson correlation analyses showed similar correlation trends in the hydrogen group, with lower abundance of H_2_-producers corresponding to higher reduction in VAS scores (*p* = 0.033 and *p* = 0.063 in VAS scores for sleep quality and mental stress, respectively. Figure 4A and B). No correlation was observed in the placebo group. In addition, the changes in OSA-MA factors 2 and 4 did not indicate meaningful relations with H_2_-producers’ abundance in either group (Fig. 4D and E). Although no interaction was detected in the STAI state from a LMM analysis, a correlation tendency was shown with r = 0.376 and *p* = 0.085 in the favorable direction (Fig. 4F).

### Abundances of hydrogen-producing bacteria explain the β-diversity cluster observed in weighted UniFrac PCoA

**Fig. 5:**
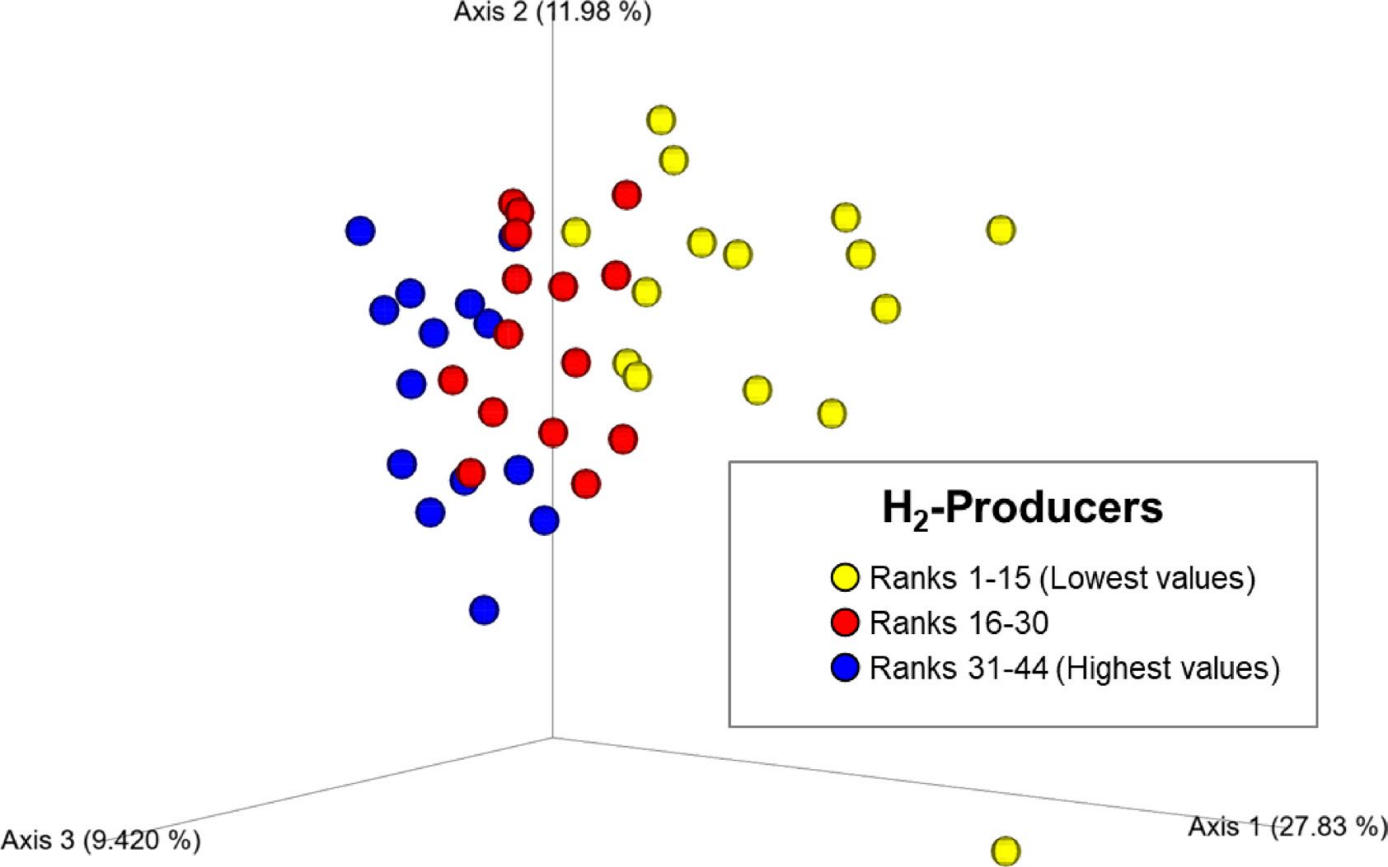
Principal coordinate analysis (PCoA) based on weighted UniFrac distances of the gut microbiome before the intervention. Dots represent individual samples and are grouped by rank according to the relative abundance of H_2_-Producers (the sum of the four hydrogen-producing bacterial genera shown in Fig. 3): yellow (lowest, ranks 1–15), red (middle, ranks 16–30), and blue (highest, ranks 31–44). Data are shown for both intervention groups before treatment.

When the relative abundance of ‘H_2_-producers’ was categorized into three levels and visualized by color in a weighted UniFrac PCoA plot, the clustering pattern, showing a pronounced gradient corresponding to the abundance of ‘H_2_-producers’, corroborated that the blue dot cluster observed in Fig. 2C reflected lower abundances of hydrogen-producing bacteria (Fig. 5).

### Influences on oxidative stress and bowel movements

Changes in oxidative stress-related outcomes and bowel movements in the overall participants are summarized in Supplementary Table S1. Serum Fe levels were significantly reduced in the hydrogen group; however, their change was not detected in the subgroup with a low relative abundance of hydrogen-producing bacteria (Supplementary Table S2). Given the considerable variability observed in the assays, these data should be regarded as supplementary reference values.

### Safety of the experimental jellies

Self-reported symptoms in daily records and clinical assessments through physical examinations and clinical questionnaires were monitored throughout the trial. No safety issues were observed with either the hydrogen-rich or placebo jelly (Supplementary Tables S3 and S4).

## Discussion

In this randomized controlled trial, we investigated the effects of an 8-week intake of hydrogen-rich jelly on sleep quality. Our prespecified primary analysis did not show significant between-group differences in changes in the sleep-related outcomes. Although subjective sleep quality improved within both groups, this may be attributed to several factors, including a strong placebo effect driven by the expectation of receiving a novel supplement, natural regression to the mean, and the Hawthorne effect due to repeated monitoring during the study.

However, exploratory analyses of the gut microbiome showed distinct β-diversity patterns associated with responsiveness to hydrogen-rich jelly. For visualization purposes, the participants were divided into two subgroups according to whether their mean changes in VAS score for sleep quality exceeded the median improvement (− 9.9 mm): ‘improved’ and ‘not improved,’ weighted UniFrac PCoA plots showed that the microbiota composition displayed a clear separation according to the improvement status in the hydrogen group but not in the placebo group. Further taxonomic analyses revealed a significant difference in the relative abundance of Bacteroides between the subgroups, based on the improvement status in the hydrogen group, which was absent in the placebo group. *Bacteroides* is a key hydrogen-producing genus that represents a substantial proportion of the total hydrogen producers in the gut^15^. Approximately 71% of gut bacterial species encode hydrogenase genes, which play a role in hydrogen production during carbohydrate fermentation. Of these, more than 85% belong to the phylum Bacteroidota or Firmicutes^13^. This is the proportion of species possessing the hydrogenase gene, and it does not directly reflect the abundance of hydrogen-producing bacteria in the gut. Considering the whole gut as one ecosystem, in which a metabolism network of inter-microbes exists, we thought that it is better to include more than one bacterial genus to determine the relationship between the benefits of exogenous hydrogen and endogenous hydrogen production capacity, although *Escherichia*, *Ruminococcus*, and *Clostridium *sensu stricto* 1* were considerably lower in relative abundance than *Bacteroides* (Fig. 3). The combined relative abundance of these four genera showed a lower *p*-value than *Bacteroides* alone between the subgroups according to the improvement status in the hydrogen group. Therefore, we used these sums as ‘H_2_-producers’ to further explore their influence on the effects of exogenous hydrogen on sleep quality.

As mentioned above, we first treated the mean change in the VAS score for sleep quality as a binary variable solely for exploratory visualization purposes. Our main interpretation regarding the relationship between baseline microbial hydrogen-producing capacity and response to hydrogen supplementation is based on continuous-variable analyses, including the LMMs and correlation analyses, rather than on the dichotomized categorization itself. Furthermore, the weighted UniFrac distance PCoA in Fig. 5 shows a gradient corresponding to the relative abundance of ‘H_2_-producers’, which is consistent with the interpretation that individuals with a lower abundance of hydrogen-producing bacteria may respond more favorably to exogenous hydrogen supplementation.

To further visualize the direction of these continuous effects, we present an additional exploratory bar plot in Supplementary Figure S1. Although the choice of a specific threshold for dichotomization in the relative abundance of ‘H_2_-producers’ is inherently arbitrary, a threshold of 0.32 was selected based on Fig. 3E, where all participants with a relative abundance below this value fell into the improved subgroup for sleep quality. This supplementary visualization is intended only to illustrate the direction of change within the intervention groups, including variables for which the LMM analysis did not show statistical significance. In the ‘H_2_-producers-Low’ subgroup (< 0.32), OSA-MA Factor 5 (sleep duration), VAS scores for sleep quality, and VAS scores for mental stress were significantly improved in the hydrogen group. These subgroup-level observations should be interpreted as descriptive and exploratory.

The individual variation in ‘H_2_-producers’ ranged from 0.1% to 57.5% (average: 34.8%) of the total gut microbes. Of these, the dominant bacteria in the gut, *Bacteroides*, also showed considerable variation, ranging from 0% to 46.2% (average: 31.7%). This suggests that substantial inter-individual variability in endogenous hydrogen-producing potential, and the hydrogen metabolism network in the gut, as well as the systemic benefits provided by microbiota-derived hydrogen, may vary tens of times or even more inter-individually.

Although our intervention was an 8-week chronic supplementation, oral hydrogen administration inherently causes transient, intermittent spikes in systemic hydrogen concentrations, which typically peak within 15 min and return to baseline within a few hours^19,20^. Previous studies demonstrate that such intermittent spikes—rather than continuous exposure—are crucial for activating hormetic pathways like Nrf2 to exert therapeutic effects^32,33^. Because the daily jelly intake in our study acts as repeated intermittent spikes, we hypothesize that the efficacy of these exogenous spikes depends heavily on the individual’s endogenous hydrogen baseline. Importantly, Suzuki et al. reported that baseline endogenous hydrogen production exhibits approximately ten-fold inter-individual variation among healthy individuals^34^. Therefore, in participants with low hydrogen-producing bacteria, the exogenous spike provides a distinct therapeutic signal over their low baseline. Conversely, in high H_2_-producers, this exogenous peak is likely masked by abundant continuous endogenous production. This “signal-to-noise” concept provides a hypothesis-generating framework that may explain the responder/non-responder patterns observed in our study.

Recent studies have shown that hydrogen administration improves sleep consolidation in mice^9^, and alleviates fatigue or improves subjective sleep quality in patients with long-COVID^10^ or obesity^11^. Since long-COVID and obesity are often associated with gut microbiota dysbiosis, such as altered Firmicutes/Bacteroidota ratios^35,36^, these populations may include a higher proportion of responders with a low abundance of hydrogen-producing bacteria. Although evidence of the underlying mechanism remains limited, these reports and our current findings suggest that exogenous hydrogen warrants further investigation as a therapeutic strategy for sleep disturbances.

The gut microbiota characteristics were stable during the intervention period in both groups. A participant in the placebo group whose *Bacteroides* at baseline was undetectable (the bottom right dot in Fig. 2B and 5) showed consistently low relative abundance of hydrogen producers, 0.016% in *Bacteroides* after intervention, and 0.086% and 0.726% in ‘H_2_-producers’ (the sum of the four genera) at baseline and after intervention, respectively. Hydrogen-rich jelly supplementation did not alter the gut microbiota composition in this study, although some studies have reported that hydrogen-rich water modifies the gut microbiota^37–39^. Exhaled hydrogen reaches a peak approximately 15 min after oral intake of hydrogen-rich water, as mentioned earlier^19,20^. The hydrogen-rich jelly used in this study was soft and could be swallowed quickly. Therefore, the absorption of hydrogen in the jelly is likely similar to that of hydrogen-rich water. In this case, exogenous hydrogen is considered to be absorbed before it reaches the colon and does not directly affect the gut microbiota.

The effects of hydrogen supplementation on sleep quality were determined not only by VAS scores but also by OSA-MA and PSQI in this study; however, the results of OSA-MA and PSQI were inconsistent with those of VAS scores. In OSA-MA, PSQI, and STAI, all or most questions were multiple-choice with four options. Therefore, a weekly questionnaire may have limited the ability to detect changes compared to the VAS, which is a continuous scale and asks for overall sleep quality or mental stress; consequently, the VAS may be more sensitive to changes. Furthermore, measures such as the PSQI and STAI are originally validated for assessing changes over several weeks rather than a 7-day period, which may further explain their limited sensitivity to weekly fluctuations in our study.

In this study, hydrogen supplementation was expected to reduce systemic oxidative stress, as reflected by decreases in markers such as 8-OHdG and isoprostanes. Although a modest reduction in 8-OHdG was observed in participants with a low relative abundance of H_2_-producers (< 0.32) in the hydrogen group, this reduction was not statistically significant, and the overall changes across oxidative damage markers, antioxidant status, and related metabolites were inconsistent. Given the inter-individual variability and possible measurement errors, these results should be interpreted with caution. Therefore, the present findings are regarded as exploratory reference values, and further studies with larger sample sizes are warranted to clarify the potential antioxidant effects of long-term hydrogen supplementation.

No safety issues were detected throughout the study period with hydrogen-rich jelly supplementation, consistent with the findings of the following studies. Liu et al. found that inhalation of 2.4% hydrogen gas did not cause clinically significant adverse effects in healthy adults, suggesting that such exposure is well tolerated^5^. Additionally, a comprehensive review by Kurokawa et al. assessed 81 clinical trials and 64 scientific publications on human studies, concluding that hydrogen therapy is nearly nontoxic across various administration methods^12^.

Hydrogen has been proposed to exert antioxidant effects and has been studied in various disease contexts; however, the baseline abundance of hydrogen producers has not been examined as a potential modifier of intervention effects. These exploratory findings raise the hypothesis that considering the gut microbiota composition, particularly the hydrogen-producing capacity, may clarify the potential benefits of exogenous hydrogen in human health and support a personalized medicine approach. Hydrogen produced by gut bacteria may be subsequently utilized by cross-feeding microbes. These hydrogen-consuming microbes are classified into three functional groups: sulfate-reducing bacteria, methanogenic archaea, and acetogenic bacteria^14^. Analysis of possible hydrogen-consuming taxa showed that their distribution did not differ according to the improvement status. The major hydrogen-consuming microbes, *Desulfovibrio* and *Methanobrevibacter,* were undetectable in most participants, and their levels were very low in the rare cases where they were detected. This suggests that the observed response pattern to exogenous hydrogen was more strongly associated with hydrogen-producing potential than hydrogen consumption by the gut microbiome.

As mentioned above, the sum of the four genera of hydrogen-producing bacteria was a useful indicator, showing a statistically significant relationship with changes in self-reported sleep quality (VAS), as indicated by the results from a LMM and Pearson correlation. Nevertheless, the relative abundance of ‘H_2_-producers’ was well correlated with that of *Bacteroides* (r = 0.905, *p* < 0.001), which is predominantly found in the gut. The correlation coefficient between the relative abundance of *Bacteroides* and the mean change in the VAS score for sleep quality was r = 0.423, with a* p*-value of 0.050 (Supplementary Figure S2). Therefore, *Bacteroides* alone may be sufficient for practically estimating responsiveness to exogenous hydrogen.

This study had several limitations. First, the sample size was relatively small, and the study population was racially homogeneous, which restricted generalizability. Second, the underlying mechanisms of exogenous hydrogen’s effect on sleep quality remain insufficiently elucidated. Third, although calorie intake was monitored during the intervention period, the background diet was not comprehensively characterized, and its possible influence on gut microbiota composition and sleep-related outcomes cannot be fully excluded. Finally, recent antibiotic use was not an exclusion criterion, and although no participant reported antibiotic use immediately before stool sampling, longer-lasting effects of prior antibiotic exposure on gut microbiota composition cannot be ruled out. To overcome these limitations, future studies should employ larger and more diverse cohorts, incorporate objective sleep measurements, further investigate mechanistic pathways, and more comprehensively assess dietary background and recent antibiotic exposure. Exhaled hydrogen monitoring will confirm the association between the biokinetics of exogenous and endogenous hydrogen and the composition of gut microbiota.

In conclusion, while the overall between-group analysis did not demonstrate statistically significant differences in the primary sleep outcomes, our findings suggest that considering gut microbiota composition may help clarify inter-individual variability in response to hydrogen supplementation and that this microbiome-informed stratification approach could be relevant beyond sleep-related outcomes. These exploratory results support the hypothesis that individuals with low microbial hydrogen-producing capacity may show greater responsiveness to hydrogen supplementation. Future clinical studies incorporating individual microbial hydrogen-producing capacity may also help clarify the mechanisms underlying hydrogen’s potential effects. If clinically meaningful cut-off values can be established, microbiome-informed stratification may help predict individual responsiveness in advance and support a more personalized approach to hydrogen-based interventions. Finally, in the present study, hydrogen-rich jelly supplementation tended to be associated with greater improvement in subjective sleep quality among individuals with a low abundance of hydrogen-producing bacteria.

## Supplementary Information

Below is the link to the electronic supplementary material.

Supplementary Material 1

## Data Availability

The datasets generated and/or analyzed during the current study are not publicly available due to ethical and privacy considerations. However, de-identified data can be shared by the corresponding author upon reasonable request, subject only to appropriate ethics approval where applicable.

## Abbreviations

CTCAE: Common terminology criteria for adverse events
OSA-MA: OSA sleep inventory MA version
PSQI: Pittsburgh sleep quality index
STAI: State-trait anxiety inventory
VAS: Visual analog scale
