# Supplementary material for: Individual variability in hydrogen-producing microbes influences the response to hydrogen supplementation on sleep quality: a randomized, double-blind, placebo-controlled, parallel study
Source: Sci Rep. 2026 Jul 27;16:23337. doi: 10.1038/s41598-026-52342-9 (PMC13407900; doi:10.1038/s41598-026-52342-9)
Supplement: Supplementary file 1 — Supplementary Material 1 [file 41598_2026_52342_MOESM1_ESM.pdf]

Supplementary Figure S1. Response to supplementation with hydrogen-rich jelly stratified by the relative abundance of the four hydrogen-producing bacterial genera shown in Figure 3.

H_2-Producers -High: Relative abundance of H_2-Producers ≥ 0.32 (n = 14 and 15 in the placebo and hydrogen groups, respectively). H_2-Producers -Low: Relative abundance of H_2-Producers < 0.32 (n = 8 and 7 in the placebo and hydrogen groups, respectively). Data are expressed as mean ± SE.

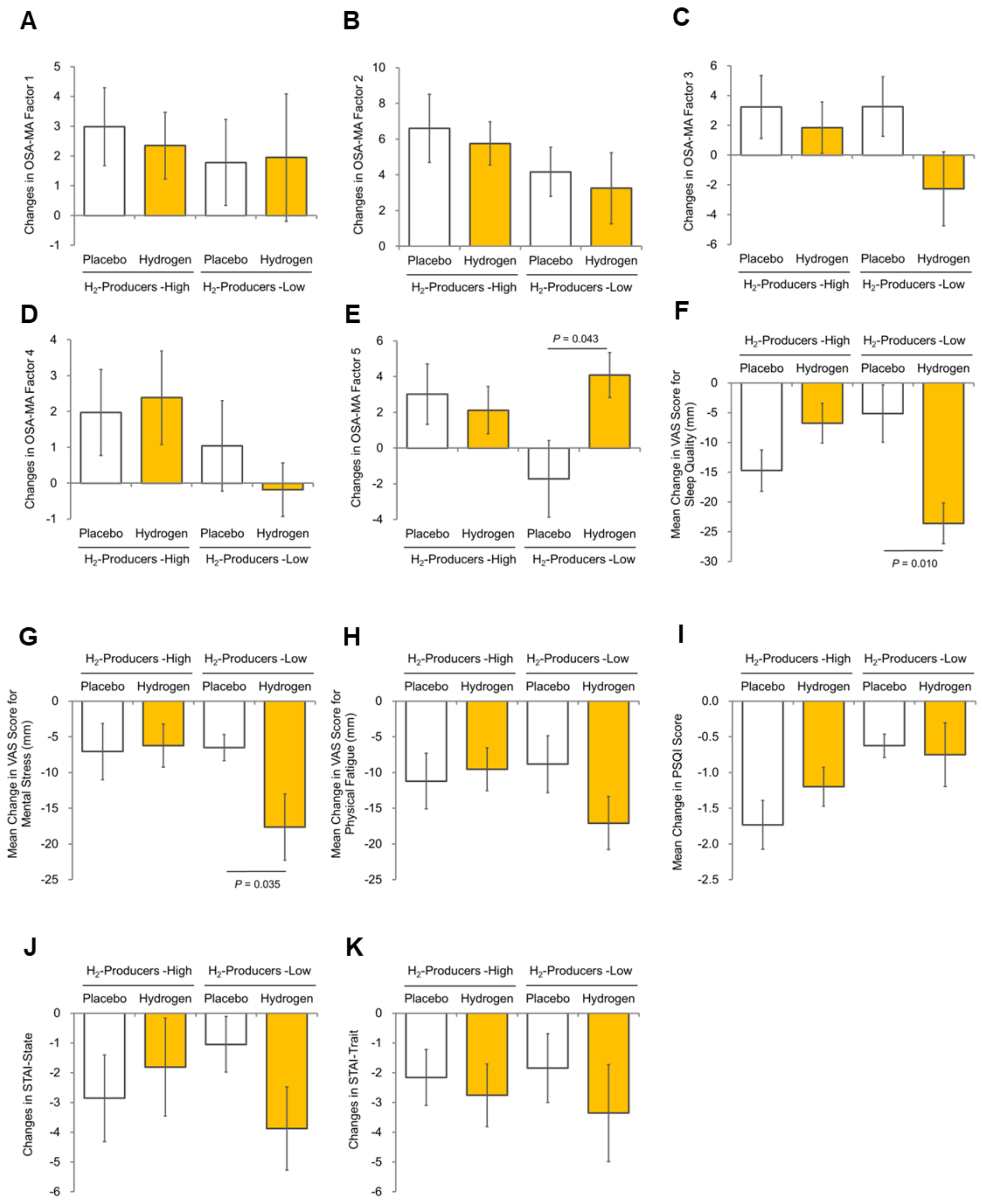

Supplementary Figure S2. Correlation between the change in each VAS score and the relative abundance of Bacteroides at baseline.

(A) VAS score for sleep quality; (B) VAS score for mental stress; (C) VAS score for physical fatigue. Pearson correlation coefficients (r) and corresponding *p*-values are shown in each plot.

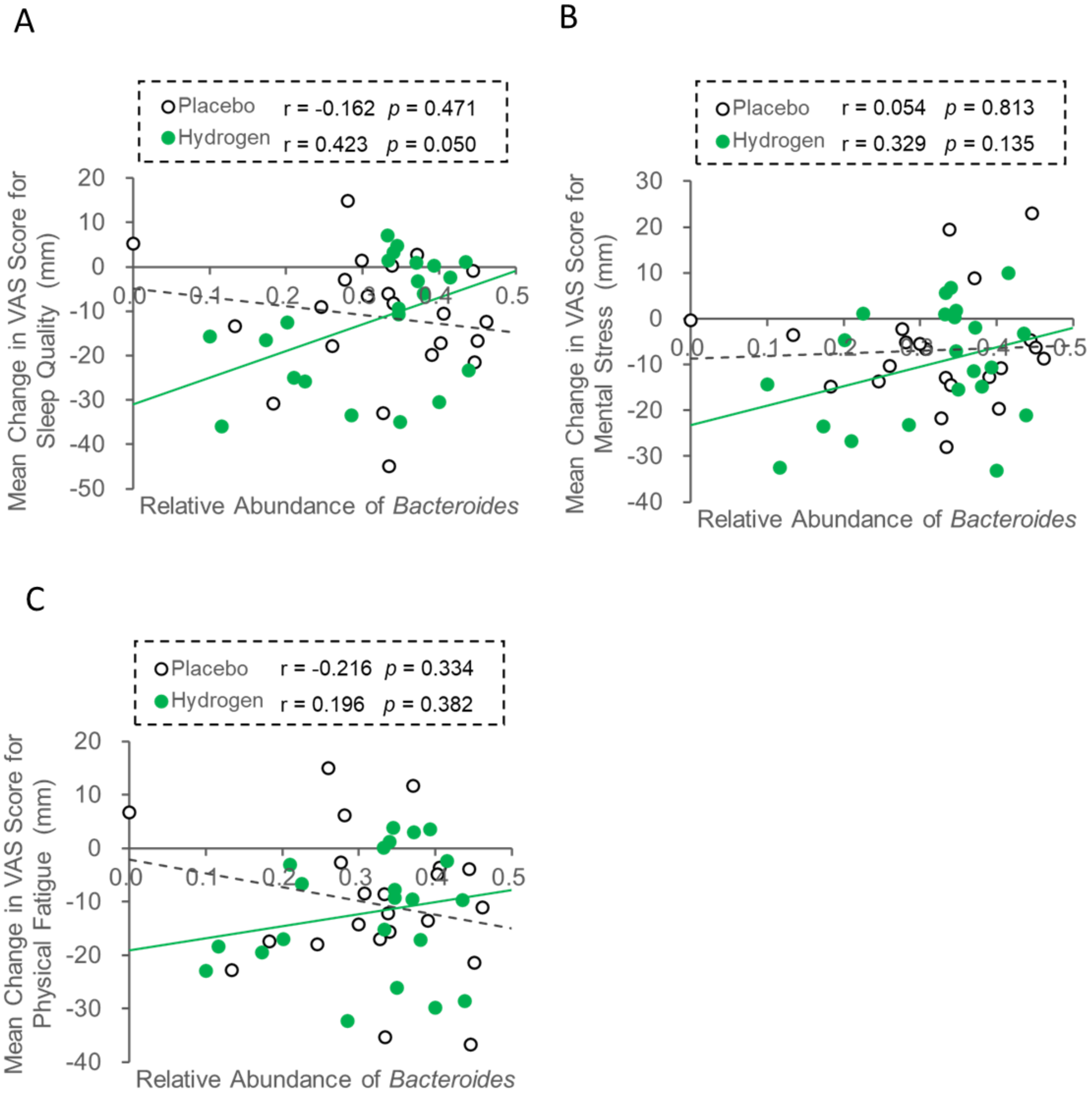

Supplementary Table S1. Changes in oxidative stress-related outcomes and bowel movements in the overall participants.

	Placebo (n = 22)			Hydrogen (n = 22)		
	week 0	week 8	Change in 8 weeks	week 0	week 8	Change in 8 weeks
Oxidative Stress Markers						
8-hydroxy-2'-deoxyguanosine (8-OHdG)	7.27 ± 2.17	6.79 ± 2.43	-0.49 ± 1.77	8.10 ± 2.45	7.98 ± 2.38	-0.13 ± 1.75
Isoprostane	3.36 ± 0.95	3.12 ± 1.25	-0.24 ± 1.62	3.38 ± 1.07	3.72 ± 1.77	0.34 ± 2.11
Lipid Peroxidation (LPO)	4.50 ± 1.20	3.54 ± 0.61 †††	-0.96 ± 1.27	5.27 ± 1.94	3.98 ± 1.19 ††	-1.29 ± 2.02
Coenzyme Q10 oxidation ratio	3.40 ± 0.77	3.86 ± 0.92 †	0.46 ± 0.90	3.42 ± 0.92	4.00 ± 1.27 †††	0.58 ± 0.63
Antioxidants						
Serum Total Antioxidant Status (STAS)	1,106 ± 99	1,098 ± 86	-8 ± 58	1,113 ± 110	1,107 ± 102	-6 ± 58
(Water-soluble Antioxidants)						
Vitamin C	10.32 ± 2.67	9.82 ± 3.09	-0.5 ± 2.25	9.57 ± 3.1	9.04 ± 2.46	-0.53 ± 2.22
Folate	11.45 ± 7.2	9.58 ± 3.9	-1.87 ± 4.78	8.91 ± 3.79	8.58 ± 3.4	-0.33 ± 2.2
Vitamin B_{12}	556 ± 251	484 ± 185 ††	-73 ± 110	454 ± 233	428 ± 168	-27 ± 124
Uric acid	5.02 ± 1.45	5.03 ± 1.26	0 ± 0.72	4.92 ± 1.56	5.03 ± 1.47	0.11 ± 0.71
(Lipid-soluble Antioxidants)						
α-Tocopherol	1646 ± 460	1658 ± 553	13 ± 281	1661 ± 411	1680 ± 647	19 ± 304
δ-Tocopherol	7.87 ± 4.24	7.25 ± 4.63	-0.62 ± 2.96	6.62 ± 1.63	7.58 ± 3.52	0.96 ± 3.16
βγ-Tocopherol	127 ± 91	124 ± 78	-3 ± 39	114 ± 46	123 ± 45	9 ± 44
α-Tocopherol/TC	7.03 ± 1.48	7.17 ± 1.63	0.14 ± 0.79	7.13 ± 1.54	7.28 ± 1.99	0.14 ± 0.87
Lutein+Zeaxanthin	46.7 ± 30.6	47.3 ± 22	0.6 ± 20.2	37 ± 14.3	39.1 ± 17.2	2.1 ± 11.9
β-Cryptoxanthin	34.6 ± 29.1	21 ± 13.4 †††	-13.6 ± 20.1	25.3 ± 23.7	17 ± 19.3 †††	-8.3 ± 8
Lycopene	12.12 ± 10.19	12.35 ± 8.79	0.23 ± 6.07	9.29 ± 4.77	10.05 ± 5.27	0.77 ± 4.65
α-Carotene	9.64 ± 10.1	8.56 ± 6.79	-1.07 ± 6.3	7.44 ± 5.02	7.55 ± 5.4	0.1 ± 1.96
β-Carotene	36.8 ± 39.4	36.3 ± 32.7	-0.5 ± 15.2	26.7 ± 20.1	27.3 ± 19.6	0.5 ± 8.2
Vitamin A	52.7 ± 17.5	54.9 ± 16.4	2.3 ± 7.3	56.3 ± 13.5	60.2 ± 16.6 †	3.8 ± 8
Pro-oxidant Metals						
Serum Fe	90 ± 32	94 ± 26	4 ± 37	129 ± 41	105 ± 30 †	-24 ± 48 *
Serum Cu	110 ± 20	107 ± 22	-3 ± 10	117 ± 22	114 ± 22	-4 ± 12
Lipid profile						
Total cholesterol (TC)	233 ± 42	229 ± 35	-4 ± 23	234 ± 36	228 ± 35	-5 ± 18
Triglycerides (TG)	115 ± 102	120 ± 112	5 ± 49	107 ± 48	104 ± 37	-2 ± 30
Bowel movements						
Frequency of spontaneous bowel movements	7.1 ± 2.8	7.4 ± 3.9	0.4 ± 3.0	7.9 ± 4.7	7.7 ± 4.2	-0.2 ± 2.3
Frequency of spontaneous bowel movements (≤ 6 times/week)	4.5 ± 1.3	4.8 ± 2.0	0.3 ± 2.5	3.5 ± 1.7	4.4 ± 2.6	0.9 ± 1.2
(Placebo: n = 8, Hydrogen: n = 7)						

Data are expressed as mean ± SD.

Statistically significant differences between groups were determined using the Student's t-test or Mann-Whitney U test, depending on the data distribution. * indicates $p < 0.05$.

†, ††, ††† indicate statistically significant differences versus the corresponding baseline, determined by paired *t*-test or paired-sample Wilcoxon signed-rank test at $p < 0.05$, $p < 0.01$, *and* $p < 0.005$, respectively.

Supplementary Table S2. Changes in oxidative stress-related outcomes in participants with low relative abundance of H_2-Producers (< 0.32).

	Placebo (n = 8)			Hydrogen (n = 7)		
	week 0	week 8	Change in 8 weeks	week 0	week 8	Change in 8 weeks
Oxidative Damage						
8-hydroxy-2'-deoxyguanosine (8-OHdG)	6.91 ± 1.92	7.08 ± 2.47	0.16 ± 2.07	8.43 ± 2.92	8.09 ± 2.44	-0.34 ± 2.06
Isoprostane	3.33 ± 0.84	2.93 ± 0.99	-0.4 ± 1.31	3.51 ± 1.16	4.18 ± 1.39	0.67 ± 2.22
Lipid Peroxidation (LPO)	5.2 ± 1.5	3.8 ± 0.7	-1.4 ± 1.7	4.8 ± 1.3	3.4 ± 0.3 †	-1.3 ± 1.1
Coenzyme Q10 oxidation ratio	3.25 ± 0.83	3.51 ± 0.82	0.26 ± 0.84	3.19 ± 0.81	3.83 ± 1.02 ††	0.64 ± 0.41
Antioxidants						
Serum Total Antioxidant Status (STAS)	1108 ± 114	1103 ± 109	-6 ± 59	1122 ± 106	1109 ± 93	-12 ± 55
(Water-soluble Antioxidants)						
Vitamin C	10.73 ± 3.59	10.76 ± 3.83	0.04 ± 2.52	10.5 ± 2.75	8.74 ± 2.43 †	-1.76 ± 1.69
Folate	13.31 ± 10.61	9.86 ± 4.2	-3.45 ± 7.12	8.47 ± 2.66	9.01 ± 1.92	0.54 ± 1.95
Vitamin B_{12}	699 ± 301	575 ± 208 †	-125 ± 108	508 ± 302	483 ± 266	-25 ± 99
Uric acid	4.99 ± 1.81	5.05 ± 1.7	0.06 ± 0.61	4.87 ± 1.37	4.96 ± 1.43	0.09 ± 0.6
(Lipid-soluble Antioxidants)						
α-Tocopherol	1584 ± 397	1576 ± 379	-8 ± 336	1583 ± 225	1523 ± 211	-60 ± 186
δ-Tocopherol	7.3 ± 5.53	6.84 ± 4.11	-0.46 ± 2.97	6.07 ± 1.02	6.56 ± 2.51	0.49 ± 3.32
βγ-Tocopherol	109 ± 68	108 ± 45	-1 ± 55	92 ± 26	98 ± 18	6 ± 35
α-Tocopherol/TC	6.93 ± 1.07	7.24 ± 1.28	0.31 ± 0.86	6.93 ± 0.85	6.73 ± 0.66	-0.2 ± 0.67
Lutein+Zeaxanthin	61.8 ± 44.8	49.9 ± 26.8	-11.9 ± 26.7	46.6 ± 12.7	46.4 ± 20.5	-0.2 ± 17.8
β-Cryptoxanthin	27.8 ± 22.2	21.2 ± 12.8	-6.6 ± 16.3	32.5 ± 35.7	25.4 ± 31.8 †	-7.1 ± 6.5
Lycopene	11.24 ± 9.84	11.06 ± 6.07	-0.18 ± 6.69	9.4 ± 4.85	10.44 ± 6.17	1.04 ± 2.51
α-Carotene	11.46 ± 15.22	7.46 ± 6.7	-4 ± 8.7	9.26 ± 7.65	10.37 ± 7.88	1.11 ± 2.66
β-Carotene	38.3 ± 52.1	30.2 ± 32	-8.2 ± 22.3	40.3 ± 25.4	38.9 ± 18.4	-1.4 ± 10.6
Vitamin A	52.4 ± 20.5	55.3 ± 16.9	2.9 ± 9.5	54.9 ± 5.2	59.5 ± 9.7	4.5 ± 10.1
Pro-oxidant Metals						
Serum Fe	98 ± 28	88 ± 20	-11 ± 19	120 ± 28	111 ± 28	-9 ± 50
Serum Cu	113 ± 24	109 ± 23	-4 ± 10	110 ± 15	105 ± 10	-5 ± 8
Lipid profile						
Total cholesterol (TC)	230 ± 52	218 ± 31	-12 ± 28	229 ± 20	226 ± 17	-3 ± 14
Triglycerides (TG)	86 ± 33	98 ± 33	12 ± 31	87 ± 29	94 ± 34	7 ± 23

Data are expressed as mean ± SD.

Statistically significant differences between groups were determined using the Student's t-test or Mann-Whitney U test, depending on the data distribution.

† and †† indicate statistically significant differences versus the corresponding baseline, determined by paired *t*-test or paired-sample Wilcoxon signed-rank test at $p < 0.05$ and $p < 0.01$, respectively.

Supplementary Table S3. Number of participants who experienced adverse events during the intervention.

	Placebo (n = 22)	Hydrogen (n = 22)	*P* value
Systolic blood pressure			1
Grade 1 to 2	1 (4.5%)	0	-
Diastolic blood pressure			1
Grade 1 to 2	0	1 (4.5%)	-

Data are presented as numbers (%). *P-values* were obtained using Fisher's exact test. Grades were assigned according to the Common Terminology Criteria for Adverse Events version 5.0 (CTCAE v5.0).

Supplementary Table S4. Frequency of self-reported symptoms during the intervention period.

	Placebo (n = 22)	Hydrogen n = 22)	*P* value
General symptoms			
Influenza-like illness	1 (4.5%)	2 (9.1%)	1
Cough, Expectoration	2 (9.1%)	3 (13.6%)	1
Rhinorrhea, Sneezing, Nasal congestion	9 (40.9%)	8 (36.4%)	1
Pharyngeal pain	4 (18.2%)	4 (18.2%)	1
Pyrexia, Chills	2 (9.1%)	1 (4.5%)	1
Anorexia	0	1 (4.5%)	1
Headache	10 (45.5%)	9 (40.9%)	1
Fatigue	7 (31.8%)	7 (31.8%)	1
Gastrointestinal disorders			
Abdominal pain, Dyspepsia, Flatulence	8 (36.4%)	10 (45.5%)	0.76
Diarrhea	6 (27.3%)	8 (36.4%)	0.75
Constipation	2 (9.1%)	2 (9.1%)	1
Nausea	0	2 (9.1%)	0.49
Pain			
Neck, shoulder, back	4 (18.2%)	1 (4.5%)	0.34
Back pain, lower	3 (13.6%)	3 (13.6%)	1
Pain in lower extremity	4 (18.2%)	1 (4.5%)	0.34
Pain in upper extremity	2 (9.1%)	0	0.49
Others	0	1 (4.5%)	1
Others			
Eye symptoms (Eye fatigue, Eye pruritus)	2 (9.1%)	3 (13.6%)	1
Skin symptoms (Rash, Pruritus)	2 (9.1%)	2 (9.1%)	1
Somnolence, Insomnia	7 (31.8%)	6 (27.3%)	1
Dizziness	1 (4.5%)	0	1
Stomatitis and other oral symptoms	1 (4.5%)	2 (9.1%)	1
Dysmenorrhea	1 (4.5%)	0	1
Psychological symptom	0	1 (4.5%)	1
Alcohol intolerance	2 (9.1%)	2 (9.1%)	1
Pernio	1 (4.5%)	0	1
Lymphadenopathy	1 (4.5%)	0	1
Peripheral edema (lower limb)	1 (4.5%)	0	1
Pollakiuria	1 (4.5%)	0	1

Data are presented as numbers (%). *P-values* were obtained using Fisher's exact test.
